# Supremum, infimum and hyperlimits in the non-Archimedean ring of Colombeau generalized numbers

**DOI:** 10.1007/s00605-021-01590-0

**Published:** 2021-07-03

**Authors:** A. Mukhammadiev, D. Tiwari, G. Apaaboah, P. Giordano

**Affiliations:** 1 University of Vienna, Wien, Austria; 2 University Grenoble Alpes, Saint-Martin-d’Héres, France

**Keywords:** Colombeau generalized numbers, Non-Archimedean rings, Generalized functions, 46F-XX, 46F30, 26E30

## Abstract

It is well-known that the notion of limit in the sharp topology of sequences of Colombeau generalized numbers $\widetilde{\mathbb {R}}$ does not generalize classical results. E.g. the sequence $\frac{1}{n}\not \rightarrow 0$ and a sequence $(x_{n})_{n\in \mathbb {N}}$ converges *if* and only if $x_{n+1}-x_{n}\rightarrow 0$. This has several deep consequences, e.g. in the study of series, analytic generalized functions, or sigma-additivity and classical limit theorems in integration of generalized functions. The lacking of these results is also connected to the fact that $\widetilde{\mathbb {R}}$ is necessarily not a complete ordered set, e.g. the set of all the infinitesimals has neither supremum nor infimum. We present a solution of these problems with the introduction of the notions of hypernatural number, hypersequence, close supremum and infimum. In this way, we can generalize all the classical theorems for the hyperlimit of a hypersequence. The paper explores ideas that can be applied to other non-Archimedean settings.

## Introduction

A key concept of non-Archimedean analysis is that extending the real field $\mathbb {R}$ into a ring containing infinitesimals and infinite numbers could eventually lead to the solution of non trivial problems. This is the case, e.g., of Colombeau theory, where nonlinear generalized functions can be viewed as set-theoretical maps on domains consisting of generalized points of the non-Archimedean ring ${\widetilde{\mathbb {R}}}$. This orientation has become increasingly important in recent years and hence it has led to the study of preliminary notions of ${\widetilde{\mathbb {R}}}$ (cf., e.g., [1–4, 11, 15–17, 25]; see below for a self-contained introduction to the ring of Colombeau generalized numbers ${\widetilde{\mathbb {R}}}$).

In particular, the sharp topology on ${\widetilde{\mathbb {R}}}$ (cf., e.g., [9, 21, 22] and below) is the appropriate notion to deal with continuity of this class of generalized functions and for a suitable concept of well-posedness. This topology necessarily has to deal with balls having infinitesimal radius $r\in {\widetilde{\mathbb {R}}}$, and thus $\frac{1}{n}\not \rightarrow 0$ if $n\rightarrow +\infty$, $n\in \mathbb {N}$, because we never have $\mathbb {R}_{>0}\ni \frac{1}{n}<r$ if *r* is infinitesimal. Another unusual property related to the sharp topology can be derived from the following inequalities (where $m\in \mathbb {N}$, $n\in \mathbb {N}_{\le m}$, $r\in {\widetilde{\mathbb {R}}}_{>0}$ is an infinitesimal number, and $\left| x_{k+1}-x_{k}\right| \le r^{2}$)

$$\begin{aligned} \left| x_{m}-x_{n}\right| \le \left| x_{m}-x_{m-1}\right| +\ldots +\left| x_{n+1}-x_{n}\right| \le (m-n)r^{2}<r, \end{aligned}$$

which imply that $(x_{n})_{n\in \mathbb {N}}\in {\widetilde{\mathbb {R}}}^{\mathbb {N}}$ is a Cauchy sequence if and only if $\left| x_{n+1}-x_{n}\right| \rightarrow 0$ (actually, this is a well-known property of every ultrametric space, cf., e.g., [14, 21]). Naturally, this has several counter-intuitive consequences (arising from differences with the classical theory) when we have to deal with the study of series, analytic generalized functions, or sigma-additivity and classical limit theorems in integration of generalized functions (cf., e.g., [12, 19, 24]).

One of the aims of the present article is to solve this kind of counter-intuitive properties so as to arrive at useful notions for the theory of generalized functions. In order to settle this problem, it is important to generalize the role of the net $(\varepsilon )$, as used in Colombeau theory, into a more general $\rho =(\rho _{\varepsilon })\rightarrow 0$ (which is called a *gauge*), and hence to generalize ${\widetilde{\mathbb {R}}}$ into some  (see Definition 1). We then introduce the set of *hypernatural numbers* asso that it is natural to expect that $\frac{1}{n}\rightarrow 0$ in the sharp topology if $n\rightarrow +\infty$ with $n$, because now *n* can also take infinite values. The notion of sequence is therefore substituted with that of *hypersequence*, as a map , where $\sigma$ is, generally speaking, another gauge. As we will see, (cf. Example 27) only in this way we are able to prove e.g. that $\frac{1}{\log n}\rightarrow 0$ in  as  but only for a suitable gauge $\sigma$ (depending on $\rho$), whereas this limit does not exist if $\sigma =\rho$.

Finally, the notions of supremum and infimum are naturally linked to the notion of limit of a monotonic (hyper)sequence. Being an ordered set,  already has a definition of, let us say, supremum as least upper bound. However, as already preliminary studied and proved by [8], this definition does not fit well with topological properties of  because generalized numbers  can actually jump as $\varepsilon \rightarrow 0^{+}$ (see Sect. 4). It is well known that in $\mathbb {R}$ we have $m=\sup (S)$ if and only if *m* is an upper bound of *S* and

1.1 $$\begin{aligned} \forall r\in \mathbb {R}_{>0}\,\exists s\in S:\ m-r\le s. \end{aligned}$$

This could be generalized into the notion of *close supremum* in , generalizing [8], that results into better topological properties, see Sect. 4. The ideas presented in the present article, which is self-contained, can surely be useful to explore similar ideas in other non-Archimedean settings, such as [5, 6, 14, 18, 23].

## The Ring of Robinson Colombeau and the hypernatural numbers

In this section, we introduce our non-Archimedean ring of scalars and its subset of hypernatural numbers. For more details and proofs about the basic notions introduced here, the reader can refer e.g. to [7, 12, 13].

As we mentioned above, in order to accomplish the theory of hyperlimits, it is important to generalize Colombeau generalized numbers by taking an arbitrary asymptotic scale instead of the usual $\rho _{\varepsilon }=\varepsilon$:

### Definition 1

Let $\rho =(\rho _{\varepsilon })\in (0,1]^{I}$ be a net such that $(\rho _{\varepsilon })\rightarrow 0$ as $\varepsilon \rightarrow 0^{+}$ (in the following, such a net will be called a *gauge*), then

(i) is called the *asymptotic gauge* generated by $\rho$.
(ii) If $\mathcal {P}(\varepsilon )$ is a property of $\varepsilon \in I$, we use the notation $\forall ^{0}\varepsilon :\,\mathcal {P}(\varepsilon )$ to denote $\exists \varepsilon _{0}\in I\,\forall \varepsilon \in (0,\varepsilon _{0}]:\,\mathcal {P}(\varepsilon )$. We can read $\forall ^{0}\varepsilon$ as *for *$\varepsilon$ *small*.
(iii) We say that a net $(x_{\varepsilon })\in \mathbb {R}^{I}$ *is *$\rho$-*moderate*, and we write $(x_{\varepsilon })\in \mathbb {R}_{\rho }$ if
  $$\begin{aligned} \exists (J_{\varepsilon })\in \mathcal {I}(\rho ):\ x_{\varepsilon }=O(J_{\varepsilon })\text { as }\varepsilon \rightarrow 0^{+}, \end{aligned}$$
  i.e., if
  $$\begin{aligned} \exists N\in \mathbb {N}\,\forall ^{0}\varepsilon :\ |x_{\varepsilon }|\le \rho _{\varepsilon }^{-N}. \end{aligned}$$
(iv) Let $(x_{\varepsilon })$, $(y_{\varepsilon })\in \mathbb {R}^{I}$, then we say that $(x_{\varepsilon })\sim _{\rho }(y_{\varepsilon })$ if
  $$\begin{aligned} \forall (J_{\varepsilon })\in \mathcal {I}(\rho ):\ x_{\varepsilon }=y_{\varepsilon }+O(J_{\varepsilon }^{-1})\text { as }\varepsilon \rightarrow 0^{+}, \end{aligned}$$
  that is if
  $$\begin{aligned} \forall n\in \mathbb {N}\,\forall ^{0}\varepsilon :\ |x_{\varepsilon }-y_{\varepsilon }|\le \rho _{\varepsilon }^{n}. \end{aligned}$$
  This is a congruence relation on the ring $\mathbb {R}_{\rho }$ of moderate nets with respect to pointwise operations, and we can hence define  which we call *Robinson-Colombeau ring of generalized numbers*. This name is justified by [7, 20]: Indeed, in [20] A. Robinson introduced the notion of moderate and negligible nets depending on an arbitrary fixed infinitesimal $\rho$ (in the framework of nonstandard analysis); independently, J.F. Colombeau, cf. e.g. [7] and references therein, studied the same concepts without using nonstandard analysis, but considering only the particular infinitesimal $(\varepsilon )$.
(v) In particular, if the gauge $\rho =(\rho _{\varepsilon })$ is non-decreasing, then we say that $\rho$ is a *monotonic gauge*. Clearly, considering a monotonic gauge narrows the class of moderate nets: e.g. if $\lim _{\varepsilon \rightarrow \frac{1}{k}}x_{\varepsilon }=+\infty$ for all $k\in \mathbb {N}_{>0}$, then $(x_{\varepsilon })\notin \mathbb {R}_{\rho }$ for any monotonic gauge $\rho$.

In the following, $\rho$ will always denote a net as in Definition 1, even if we will sometimes omit the dependence on the infinitesimal $\rho$, when this is clear from the context. We will also use other directed sets instead of *I*: e.g. $J\subseteq I$ such that 0 is a closure point of *J*, or $I\times \mathbb {N}$. The reader can easily check that all our constructions can be repeated in these cases.

We also recall that we write $[x_{\varepsilon }]\le [y_{\varepsilon }]$ if there exists $(z_{\varepsilon })\in \mathbb {R}^{I}$ such that $(z_{\varepsilon })\sim _{\rho }0$ (we then say that $(z_{\varepsilon })$ is $\rho$-*negligible*) and $x_{\varepsilon }\le y_{\varepsilon }+z_{\varepsilon }$ for $\varepsilon$ small. Equivalently, we have that $x\le y$ if and only if there exist representatives $[x_{\varepsilon }]=x$ and $[y_{\varepsilon }]=y$ such that $x_{\varepsilon }\le y_{\varepsilon }$ for all $\varepsilon$.

Although the order $\le$ is not total, we still have the possibility to define the infimum $[x_{\varepsilon }]\wedge [y_{\varepsilon }]\,{:=}\,[\min (x_{\varepsilon },y_{\varepsilon })]$, the supremum $[x_{\varepsilon }]\vee [y_{\varepsilon }]\,{:=}\,\left[ \max (x_{\varepsilon },y_{\varepsilon })\right]$ of a finite number of generalized numbers. Henceforth, we will also use the customary notation  for the set of invertible generalized numbers, and we write $x<y$ to say that $x\le y$ and . Our notations for intervals are: , $[a,b]_{\mathbb {R}}\,{:=}\,[a,b]\cap \mathbb {R}$. Finally, we set , which is a positive invertible infinitesimal, whose reciprocal is , which is necessarily a strictly positive infinite number.

The following result is useful to deal with positive and invertible generalized numbers. For its proof, see e.g. [2, 3, 12, 13].

### Lemma 2

Let . Then the following are equivalent:

(i) *x* is invertible and $x\ge 0$, i.e. $x>0$.
(ii) For each representative $(x_{\varepsilon })\in \mathbb {R}_{\rho }$ of *x* we have $\forall ^{0}\varepsilon :\ x_{\varepsilon }>0$.
(iii) For each representative $(x_{\varepsilon })\in \mathbb {R}_{\rho }$ of *x* we have $\exists m\in \mathbb {N}\,\forall ^{0}\varepsilon :\ x_{\varepsilon }>\rho _{\varepsilon }^{m}$.
(iv) There exists a representative $(x_{\varepsilon })\in \mathbb {R}_{\rho }$ of *x* such that $\exists m\in \mathbb {N}\,\forall ^{0}\varepsilon :\ x_{\varepsilon }>\rho _{\varepsilon }^{m}$.

### The language of subpoints

The following simple language allows us to simplify some proofs using steps recalling the classical real field $\mathbb {R}$. We first introduce the notion of *subpoint*:

#### Definition 3

For subsets *J*, $K\subseteq I$ we write $K\subseteq _{0}J$ if 0 is an accumulation point of *K* and $K\subseteq J$ (we read it as: *K* *is co-final in J*). Note that for any $J\subseteq _{0}I$, the constructions introduced so far in Definition 1 can be repeated using nets $(x_{\varepsilon })_{\varepsilon \in J}$. We indicate the resulting ring with the symbol . More generally, no peculiar property of $I=(0,1]$ will ever be used in the following, and hence all the presented results can be easily generalized considering any other directed set. If $K\subseteq _{0}J$,  and , then $x'$ is called a *subpoint* of *x*, denoted as $x'\subseteq x$, if there exist representatives $(x_{\varepsilon })_{\varepsilon \in J}$, $(x'_{\varepsilon })_{\varepsilon \in K}$ of *x*, $x'$ such that $x'_{\varepsilon }=x_{\varepsilon }$ for all $\varepsilon \in K$. In this case we write $x'=x|_{K}$, $\text {dom}(x')\,{:=}\,K$, and the restriction  is a well defined operation. In general, for  we set .

In the next definition, we introduce binary relations that hold only *on subpoints*. Clearly, this idea is inherited from nonstandard analysis, where cofinal subsets are always taken in a fixed ultrafilter.

#### Definition 4

Let *x*, , $L\subseteq _{0}I$, then we say

(i) $x<_{L}y\ :\iff x|_{L}<y|_{L}$ (the latter inequality has to be meant in the ordered ring ). We read $x<_{L}y$ as “*x is less than y on L*”.
(ii) $x<_{\mathrm{s}}y\ :\iff \ \exists L\subseteq _{0}I:\ x<_{L}y$. We read $x<_{\mathrm{s}}y$ as “*x is less than y on subpoints”.*

Analogously, we can define other relations holding only on subpoints such as e.g.: $\in _{\mathrm{s}}$, $\le _{\mathrm{s}}$, $=_{\mathrm{s}}$, $\subseteq _{\mathrm{s}}$, etc.

For example, we have

$$\begin{aligned} x\le y\&\iff \ \forall L\subseteq _{0}I:\ x\le _{L}y\\ x<y\&\iff \ \forall L\subseteq _{0}I:\ x<_{L}y, \end{aligned}$$

the former following from the definition of $\le$, whereas the latter following from Lemma 2. Moreover, if $\mathcal {P}\left\{ x_{\varepsilon }\right\}$ is an arbitrary property of $x_{\varepsilon }$, then

2.1 $$\begin{aligned} \lnot \left( \forall ^{0}\varepsilon :\ \mathcal {P}\left\{ x_{\varepsilon }\right\} \right) \ \iff \ \exists L\subseteq _{0}I\,\forall \varepsilon \in L:\ \lnot \mathcal {P}\left\{ x_{\varepsilon }\right\} . \end{aligned}$$

Note explicitly that, generally speaking, relations on subpoints, such as $\le _{\mathrm{s}}$ or $=_{\mathrm{s}}$, do not inherit the same properties of the corresponding relations for points. So, e.g., both $=_{\mathrm{s}}$ and $\le _{\mathrm{s}}$ are not transitive relations.

The next result clarifies how to equivalently write a negation of an inequality or of an equality using the language of subpoints.

#### Lemma 5

Let *x*, , then

(i) $$x\nleq y\quad \Longleftrightarrow \quad x>_{\mathrm{s}}y$$
(ii) $$x\not <y\quad \Longleftrightarrow \quad x\ge _{\mathrm{s}}y$$
(iii) $$x\ne y\quad \Longleftrightarrow \quad x>_{\mathrm{s}}y$$ or $$x<_{\mathrm{s}}y$$

#### Proof

(i) $\Leftarrow$: The relation $x>_{\mathrm{s}}y$ means $x|_{L}>y|_{L}$ for some $L\subseteq _{0}I$. By Lemma 2 for the ring , we get $\forall ^{0}\varepsilon \in L:\ x_{\varepsilon }>y_{\varepsilon }$, where $x=[x_{\varepsilon }]$, $y=[y_{\varepsilon }]$ are any representatives of *x*, *y* resp. The conclusion follows by (2.1)

(i) $\Rightarrow$: Take any representatives $x=[x_{\varepsilon }]$, $y=[y_{\varepsilon }]$. The property

$$\begin{aligned} \forall q\in \mathbb {R}_{>0}\,\forall ^{0}\varepsilon :\ x_{\varepsilon }\le y_{\varepsilon }+\rho _{\varepsilon }^{q} \end{aligned}$$

for $q\rightarrow +\infty$ implies $x\le y$. We therefore have

$$\begin{aligned} \exists q\in \mathbb {R}_{>0}\,\exists L\subseteq _{0}I\,\forall \varepsilon \in L:\ x_{\varepsilon }>y_{\varepsilon }+\rho _{\varepsilon }^{q}, \end{aligned}$$

i.e. $$x>_{L}y$$.

(ii) $\Rightarrow$: We have two cases: either $x-y$ is not invertible or $x\not \le y$. In the former case, the conclusion follows from [13, Thm. 1.2.39]. In the latter one, it follows from (i).

(ii) $\Leftarrow$: By contradiction, if $x<y$ then $x=_{L}y$ for some $L\subseteq _{0}I$, which contradicts the invertibility of $x-y$.

(iii) $\Rightarrow$: By contradiction, assume that $x\not >_{\mathrm{s}}y$ and $x\not <_{\mathrm{s}}y$. Then (i) would yield $x\le y$ and $y\le x$, and hence $x=y$. The opposite implication directly follows by contradiction. $\square$

Using the language of subpoints, we can write different forms of dichotomy or trichotomy laws for inequality. The first form is the following

#### Lemma 6

Let *x*, , then

(i) $$x\le y$$ or $$x>_{\mathrm{s}}y$$
(ii) $$\lnot (x>_{\mathrm{s}}y$$ and $$x\le y)$$
(iii) $x=y$ or $x<_{\mathrm{s}}y$ or $x>_{\mathrm{s}}y$
(iv) $$x\le y\ \Rightarrow \ x<_{\mathrm{s}}y$$ or $$x=y$$
(v) $$x\le _{\mathrm{s}}y\ \Longleftrightarrow \ x<_{\mathrm{s}}y$$ or $$x=_{\mathrm{s}}y$$.

#### Proof

(i) and (ii) follows directly from Lemma 5. To prove (iii), we can consider that $x>_{\mathrm{s}}y$ or $x\not >_{\mathrm{s}}y$. In the second case, Lemma 5 implies $x\le y$. If $y\le x$ then $x=y$; otherwise, once again by Lemma 5, we get $x<_{\mathrm{s}}y$. To prove (iv), assume that $x\le y$ but $x\not <_{\mathrm{s}}y$, then $x\ge y$ by Lemma 5.(i) and hence $x=y$. The implication $\Leftarrow$ of (v) is trivial. On the other hand, if $x\le _{\mathrm{s}}y$ and $x\not <_{\mathrm{s}}y$, then $y\le x$ from Lemma 5.(i), and hence $x=_{\mathrm{s}}y$. $\square$

As usual, we note that these results can also be trivially repeated for the ring . So, e.g., we have $x\not \le _{L}y$ if and only if $\exists J\subseteq _{0}L:\ x>_{J}y$, which is the analog of Lemma 5.(i) for the ring .

The second form of trichotomy (which for  can be more correctly named as *quadrichotomy*)* is* stated as follows:

#### Lemma 7

Let $$x=[x_{\varepsilon }]$$, , then

(i) $$x\le y$$ or $$x\ge y$$ or $$\exists L\subseteq _{0}I:\ L^{c}\subseteq _{0}I,\ x\ge _{L}y\text { and }x\le _{L^{c}}y$$
(ii) If for all $L\subseteq _{0}I$ the following implication holds
  2.2 $$\begin{aligned} x\le _{L}y,\text { or }x\ge _{L}y\ \Rightarrow \ \forall ^{0}\varepsilon \in L:\ \mathcal {P}\left\{ x_{\varepsilon },y_{\varepsilon }\right\} , \end{aligned}$$
  then $$\forall ^{0}\varepsilon :\ \mathcal {P}\left\{ x_{\varepsilon },y_{\varepsilon }\right\} $$.
(iii) If for all $L\subseteq _{0}I$ the following implication holds
  2.3 $$\begin{aligned} x<_{L}y,\text { or }x>_{L}y\text { or }x=_{L}y\ \Rightarrow \ \forall ^{0}\varepsilon \in L:\ \mathcal {P}\left\{ x_{\varepsilon },y_{\varepsilon }\right\} , \end{aligned}$$
  then $$\forall ^{0}\varepsilon :\ \mathcal {P}\left\{ x_{\varepsilon },y_{\varepsilon }\right\} $$.

#### Proof

(i) : if $x\not \le y$, then $x>_{\mathrm{s}}y$ from Lemma 5.(i). Let $[x_{\varepsilon }]=x$ and $[y_{\varepsilon }]=y$ be two representatives, and set . The relation $x>_{\mathrm{s}}y$ implies that $L\subseteq _{0}I$. Clearly, $x\ge _{L}y$ (but note that in general we cannot prove $x>_{L}y$). If $L^{c}\not \subseteq _{0}I$, then $(0,\varepsilon _{o}]\subseteq L$ for some $\varepsilon _{0}$, i.e. $x\ge y$. On the contrary, if $L^{c}\subseteq _{0}I$, then $x\le _{L^{c}}y$.

(ii): Property (i) states that we have three cases. If $x_{\varepsilon }\le y_{\varepsilon }$ for all $\varepsilon \le \varepsilon _{0}$, then it suffices to set $L\,{:=}\,(0,\varepsilon _{0}]$ in (2.2) to get the claim. Similarly, we can proceed if $x\ge y$. Finally, if $x\ge _{L}y$ and $x\le _{L^{c}}y$, then we can apply (2.2) both with *L* and $L^{c}$ to obtain

$$\begin{aligned} \forall ^{0}\varepsilon&\in L:\ \mathcal {P}\left\{ x_{\varepsilon },y_{\varepsilon }\right\} \\ \forall ^{0}\varepsilon&\in L^{c}:\ \mathcal {P}\left\{ x_{\varepsilon },y_{\varepsilon }\right\} , \end{aligned}$$

from which the claim directly follows.

(iii): By contradiction, assume

2.4 $$\begin{aligned} \forall \varepsilon \in L:\ \lnot \mathcal {P}\left\{ x_{\varepsilon },y_{\varepsilon }\right\} , \end{aligned}$$

for some $L\subseteq _{0}I$. We apply (i) to the ring  to obtain the following three cases:

2.5 $$\begin{aligned} x\le _{L}y\text { or }x\ge _{L}y\text { or }\exists J\subseteq _{0}L:\ J^{c}\subseteq _{0}L,\ x\ge _{J}y\text { and }x\le _{J^{c}}y. \end{aligned}$$

If $x\le _{L}y$, by Lemma 6.(iv) for the ring , this case splits into two sub-cases: $x=_{L}y$ or $\exists K\subseteq _{0}L:\ x<_{K}y$. If the former holds, using (2.3) we get $\mathcal {P}\left\{ x_{\varepsilon },y_{\varepsilon }\right\}$ $\forall ^{0}\varepsilon \in L$, which contradicts (2.4). If $x<_{K}y$, then $K\subseteq _{0}I$ and we can apply (2.4) with *K* to get $\mathcal {P}\left\{ x_{\varepsilon },y_{\varepsilon }\right\}$ $\forall ^{0}\varepsilon \in K$, which again contradicts (2.4) because $K\subseteq _{0}L$. Similarly we can proceed with the other three cases stated in (2.5). $\square$

Property Lemma 7.(ii) represents a typical replacement of the usual dichotomy law in $\mathbb {R}$: for arbitrary $L\subseteq _{0}I$, we can assume to have two cases: either $x\le _{L}y$ or $x\ge _{L}y$. If in both cases we are able to prove $\mathcal {P}\{x_{\varepsilon },y_{\varepsilon }\}$ for $\varepsilon \in L$ small, then we always get that this property holds for all $\varepsilon$ small. Similarly, we can use the strict trichotomy law stated in (iii).

### Inferior, superior and standard parts

Other simple tools that we can use to study generalized numbers of  are the *inferior *and *superior parts *of a number. Only in this section of the article, we assume that $\rho$ is a monotonic gauge.

#### Definition 8

Let  be a generalized number, then:

(i) If $\exists L\in \mathbb {R}:\ L\le x$, then $x_{\text {i}}\,{:=}\,\left[ \inf _{e\in (0,\varepsilon ]}x_{e}\right]$ is called the *inferior part of x.*
(ii) If $\exists U\in \mathbb {R}:\ x\le U$, then $x_{\text {s}}\,{:=}\,\left[ \sup _{e\in (0,\varepsilon ]}x_{e}\right]$ is called the *superior part of x.*

Moreover, we set:

(iii) ${x_{\text {i}}^\circ }\,{:=}\,\liminf _{\varepsilon \rightarrow 0^{+}}x_{\varepsilon }\in \mathbb {R}\cup \{\pm \infty \}$, where $[x_{\varepsilon }]=x$ is any representative of *x*, is called the *inferior standard part* of *x*. Note that if $\exists x_{\text {i}}$, i.e. if *x* is finitely bounded from below, then ${(x_{\text {i}})^\circ }={x_{\text {i}}^\circ }\in \mathbb {R}$ and ${x_{\text {i}}^\circ }\ge x_{\text {i}}$.
(iv) ${x_{\text {s}}^\circ }\,{:=}\,\limsup _{\varepsilon \rightarrow 0^{+}}x_{\varepsilon }\in \mathbb {R}\cup \{\pm \infty \}$, where $[x_{\varepsilon }]=x$ is any representative of *x*, is called the *superior standard part* of *x*. Note that if $\exists x_{\text {s}}$, i.e. if *x* is finitely bounded from above, then ${(x_{\text {s}})^\circ }={x_{\text {s}}^\circ }\in \mathbb {R}$ and ${x_{\text {s}}^\circ }\le x_{\text {s}}$.

Note that, since $\rho =(\rho _{\varepsilon })$ is non-decreasing, if $[x'_{\varepsilon }]=x$ is another representative, then for all $e\in (0,\varepsilon ]$, we have $x'_{e}\le x_{e}+\rho _{e}^{n}\le x_{e}+\rho _{\varepsilon }^{n}\le \rho _{\varepsilon }^{n}+\sup _{e\in (0,\varepsilon ]}x_{e}$ and hence $\sup _{e\in (0,\varepsilon ]}x'_{e}\le \rho _{\varepsilon }^{n}+\sup _{e\in (0,\varepsilon ]}x_{e}$. This shows that inferior and superior parts, when they exist, are well-defined. Moreover, if $(z_{\varepsilon })$ is negligible, then $\limsup _{\varepsilon \rightarrow 0^{+}}\left( x_{\varepsilon }+z_{\varepsilon }\right) \le \limsup _{\varepsilon \rightarrow 0^{+}}x_{\varepsilon }+0$, which shows that ${x_{\text {s}}^\circ }$ is well-defined (similarly for ${x_{\text {i}}^\circ }$ using super-additivitiy of $\liminf$).

Clearly, $x_{\text {i}}\le x\le x_{\text {s}}$ and ${x_{\text {i}}^\circ }\le {x_{\text {s}}^\circ }$. We have that the generalized number *x* is near-standard if and only if ${x_{\text {i}}^\circ }={x_{\text {s}}^\circ }{=}{:}{x^\circ }\in \mathbb {R}$; it is infinitesimal if and only if $\exists \,{x^\circ }=0$; it is a positive infinite number if and only if ${x_{\text {i}}^\circ }={x_{\text {s}}^\circ }{=}{:}{x^\circ }=+\infty$ (the same for negative infinite numbers); it is a finite number if and only if ${x_{\text {i}}^\circ },$ ${x_{\text {s}}^\circ }\in \mathbb {R}$. Finally, there always exist $x'$, $x''\subseteq x$ such that $x'\approx {x_{\text {i}}^\circ }$ and $x''\approx {x_{\text {s}}^\circ }$, where $x\approx y$ means that $x-y$ is infinitesimal (i.e. $|x-y|\le r$ for all $r\in \mathbb {R}_{>0}$ or, equivalently, $\lim _{\varepsilon \rightarrow 0^{+}}x_{\varepsilon }-y_{\varepsilon }=0$ for all $[x_{\varepsilon }]=x$, $[y_{\varepsilon }]=y$). Therefore, any generalized number in  is either finite or some of its subpoints is infinite; in the former case, some of its subpoints is near standard.

### Topologies on 

On the -module  we can consider the natural extension of the Euclidean norm, i.e. , where . Even if this generalized norm takes values in , it shares some essential properties with classical norms:

$$\begin{aligned}&|x|=x\vee (-x)\\&|x|\ge 0\\&|x|=0\Rightarrow x=0\\&|y\cdot x|=|y|\cdot |x|\\&|x+y|\le |x|+|y|\\&||x|-|y||\le |x-y|. \end{aligned}$$

It is therefore natural to consider on  topologies generated by balls defined by this generalized norm and a set of radii:

#### Definition 9

We say that $\mathfrak {R}$ is a *set of radii* if

(i) is a non-empty subset of positive invertible generalized numbers.
(ii) For all *r*, $s\in \mathfrak {R}$ the infimum $r\wedge s\in \mathfrak {R}$.
(iii) $k\cdot r\in \mathfrak {R}$ for all $r\in \mathfrak {R}$ and all $k\in \mathbb {R}_{>0}$.

Moreover, if $\mathfrak {R}$ is a set of radii and *x*, , then:

(i) We write $x<_{\mathfrak {R}}y$ if $\exists r\in \mathfrak {R}:\ r\le y-x$.
(ii) for any $r\in \mathfrak {R}$.
(iii), for any $\rho \in \mathbb {R}_{>0}$, denotes an ordinary Euclidean ball in $\mathbb {R}^{n}$.

For example,  and $\mathbb {R}_{>0}$ are sets of radii.

#### Lemma 10

Let $\mathfrak {R}$ be a set of radii and *x*, *y*, , then

(i) $$\lnot \left( x<_{\mathfrak {R}}x\right) $$.
(ii) $x<_{\mathfrak {R}}y$ and $y<_{\mathfrak {R}}z$ imply $x<_{\mathfrak {R}}z$.
(iii) $$\forall r\in \mathfrak {R}:\ 0<_{\mathfrak {R}}r$$.

The relation $<_{\mathfrak {R}}$ has better topological properties as compared to the usual strict order relation $x\le y$ and $x\ne y$ (a relation that we will therefore never use) because of the following result:

#### Theorem 11

The set of balls  generated by a set of radii $\mathfrak {R}$ is a base for a topology on .

Henceforth, we will only consider the sets of radii  and $\mathbb {R}_{>0}$ and will use the simplified notation $B_{r}(x)\,{:=}\,B_{r}^{\mathfrak {\mathfrak {R}}}(x)$ if . The topology generated in the former case is called *sharp topology*, whereas the latter is called *Fermat topology*. We will call *sharply open set* any open set in the sharp topology, and *large open set* any open set in the Fermat topology; clearly, the latter is coarser than the former. It is well-known (see e.g. [2, 3, 9, 10, 12] and references therein) that this is an equivalent way to define the sharp topology usually considered in the ring of Colombeau generalized numbers. We therefore recall that the sharp topology on  is Hausdorff and Cauchy complete, see e.g. [2, 10].

### Open, closed and bounded sets generated by nets

A natural way to obtain sharply open, closed and bounded sets in  is by using a net $(A_{\varepsilon })$ of subsets $A_{\varepsilon }\subseteq \mathbb {R}^{n}$. We have two ways of extending the membership relation $x_{\varepsilon }\in A_{\varepsilon }$ to generalized points  (cf. [11, 17]):

#### Definition 12

Let $(A_{\varepsilon })$ be a net of subsets of $\mathbb {R}^{n}$, then

(i) is called the *internal set* generated by the net $(A_{\varepsilon })$.
(ii) Let $(x_{\varepsilon })$ be a net of points of $\mathbb {R}^{n}$, then we say that $x_{\varepsilon }\in _{\varepsilon }A_{\varepsilon }$, and we read it as $(x_{\varepsilon })$ *strongly belongs to *$(A_{\varepsilon })$, if
  (i) $$\forall ^{0}\varepsilon :\ x_{\varepsilon }\in A_{\varepsilon }$$.
  (ii) If $(x'_{\varepsilon })\sim _{\rho }(x_{\varepsilon })$, then also $x'_{\varepsilon }\in A_{\varepsilon }$ for $\varepsilon$ small.
  Moreover, we set , and we call it the *strongly internal set* generated by the net $(A_{\varepsilon })$.
(iii) We say that the internal set $K=[A_{\varepsilon }]$ is *sharply bounded* if there exists  such that $K\subseteq B_{M}(0)$.
(iv) Finally, we say that the net $(A_{\varepsilon })$ is *sharply bounded *if there exists $N\in \mathbb {R}_{>0}$ such that $\forall ^{0}\varepsilon \,\forall x\in A_{\varepsilon }:\ |x|\le \rho _{\varepsilon }^{-N}$.

Therefore, $x\in [A_{\varepsilon }]$ if there exists a representative $[x_{\varepsilon }]=x$ such that $x_{\varepsilon }\in A_{\varepsilon }$ for $\varepsilon$ small, whereas this membership is independent from the chosen representative in case of strongly internal sets. An internal set generated by a constant net $A_{\varepsilon }=A\subseteq \mathbb {R}^{n}$ will simply be denoted by [*A*].

The following theorem (cf. [11, 17] for the case $\rho _{\varepsilon }=\varepsilon$, and [12] for an arbitrary gauge) shows that internal and strongly internal sets have dual topological properties:

#### Theorem 13

For $\varepsilon \in I$, let $A_{\varepsilon }\subseteq \mathbb {R}^{n}$ and let $x_{\varepsilon }\in \mathbb {R}^{n}$. Then we have

(i) $[x_{\varepsilon }]\in [A_{\varepsilon }]$ if and only if $\forall q\in \mathbb {R}_{>0}\,\forall ^{0}\varepsilon :\ d(x_{\varepsilon },A_{\varepsilon })\le \rho _{\varepsilon }^{q}$. Therefore $[x_{\varepsilon }]\in [A_{\varepsilon }]$ if and only if .
(ii) $[x_{\varepsilon }]\in \langle A_{\varepsilon }\rangle$ if and only if $\exists q\in \mathbb {R}_{>0}\,\forall ^{0}\varepsilon :\ d(x_{\varepsilon },A_{\varepsilon }^{\text {c}})>\rho _{\varepsilon }^{q}$, where $A_{\varepsilon }^{\text {c}}\,{:=}\,\mathbb {R}^{n}\setminus A_{\varepsilon }$. Therefore, if $(d(x_{\varepsilon },A_{\varepsilon }^{\text {c}}))\in \mathbb {R}_{\rho }$, then $[x_{\varepsilon }]\in \langle A_{\varepsilon }\rangle$ if and only if $[d(x_{\varepsilon },A_{\varepsilon }^{\text {c}})]>0$.
(iii) $[A_{\varepsilon }]$ is sharply closed.
(iv) $\langle A_{\varepsilon }\rangle$ is sharply open.
(v) $[A_{\varepsilon }]=\left[ {\varvec{{cl}}}\left( A_{\varepsilon }\right) \right]$, where ${\varvec{{cl}}}\left( S\right)$ is the closure of $S\subseteq \mathbb {R}^{n}$.
(vi) $\langle A_{\varepsilon }\rangle =\langle {\varvec{{int}\left( A_{\varepsilon }\right) }}\rangle$, where $int \left( S\right)$ is the interior of $S\subseteq \mathbb {R}^{n}$.

For example, it is not hard to show that the closure in the sharp topology of a ball of center $c=[c_{\varepsilon }]$ and radius $r=[r_{\varepsilon }]>0$ is2.6whereasUsing internal sets and adopting ideas similar to those used in proving Lemma 7, we also have the following form of dichotomy law:

#### Lemma 14

For $\varepsilon \in I$, let $A_{\varepsilon }\subseteq \mathbb {R}^{n}$ and let . Then we have:

(i) $$x\in [A_{\varepsilon }]$$ or $$x\in [A_{\varepsilon }^{c}]$$ or $$\exists L\subseteq _{0}I:\ L^{c}\subseteq _{0}I,\ x\in _{L}[A_{\varepsilon }],\ x\in _{L^{c}}[A_{\varepsilon }^{c}]$$
(ii) If for all $L\subseteq _{0}I$ the following implication holds
  $$\begin{aligned} x\in _{L}[A_{\varepsilon }]\text { or }x\in _{L}[A_{\varepsilon }^{c}]\ \Rightarrow \ \forall ^{0}\varepsilon \in L:\ \mathcal {P}\{x_{\varepsilon }\}, \end{aligned}$$
  then $$\forall ^{0}\varepsilon :\ \mathcal {P}\{x_{\varepsilon }\}$$.

#### Proof

(i): If $x\notin [A_{\varepsilon }^{c}]$, then $x_{\varepsilon }\in A_{\varepsilon }$ for all $\varepsilon \in K$ and for some $K\subseteq _{0}I$. Set , so that $K\subseteq L\subseteq _{0}I$. We have $x\in _{L}[A_{\varepsilon }]$. If $L^{c}\not \subseteq _{0}I$, then $(0,\varepsilon _{0}]\subseteq L$ for some $\varepsilon _{0}$, i.e. $x\in [A_{\varepsilon }]$. On the contrary, if $L^{c}\subseteq _{0}I$, then $x\in _{L^{c}}[A_{\varepsilon }^{c}]$.

(ii): We can proceed as in the proof of Lemma 7.(ii) using (i). $\square$

## Hypernatural numbers

We start by defining the set of hypernatural numbers in  and the set of $\rho$-moderate nets of natural numbers:

### Definition 15

We set

(i)
(ii)

Therefore,  if and only if there exists $(x_{\varepsilon })\in \mathbb {R}_{\rho }$ such that $n=[\text {int}(|x_{\varepsilon }|)]$. Clearly, . Note that the integer part function $\text {int}(-)$ is not well-defined on . In fact, if $x=1=\left[ 1-\rho _{\varepsilon }^{1/\varepsilon }\right] =\left[ 1+\rho _{\varepsilon }^{1/\varepsilon }\right]$, then $\text {int}\left( 1-\rho _{\varepsilon }^{1/\varepsilon }\right) =0$ whereas $\text {int}\left( 1+\rho _{\varepsilon }^{1/\varepsilon }\right) =1$, for $\varepsilon$ sufficiently small. Similar counter examples can be set for floor and ceiling functions. However, the nearest integer function is well defined on , as proved in the following

### Lemma 16

Let $(n_{\varepsilon })\in \mathbb {N}_{\rho }$ and $(x_{\varepsilon })\in \mathbb {R}_{\rho }$ be such that $[n_{\varepsilon }]=[x_{\varepsilon }]$. Let $\text {rpi }:\mathbb {R}\longrightarrow \mathbb {N}$ be the function rounding to the nearest integer with tie breaking towards positive infinity, i.e. $\text {rpi}(x)=\lfloor x+\frac{1}{2}\rfloor$. Then $\text {rpi }(x_{\varepsilon })=n_{\varepsilon }$ for $\varepsilon$ small. The same result holds using $\text {rni }:\mathbb {R}\longrightarrow \mathbb {N}$, the function rounding half towards $-\infty$.

### Proof

We have $\text {rpi}(x)=\lfloor x+\frac{1}{2}\rfloor$, where $\lfloor -\rfloor$ is the floor function. For $\varepsilon$ small, $\rho _{\varepsilon }<\frac{1}{2}$ and, since $[n_{\varepsilon }]=[x_{\varepsilon }]$, always for $\varepsilon$ small, we also have $n_{\varepsilon }-\rho _{\varepsilon }+\frac{1}{2}<x_{\varepsilon }+\frac{1}{2}<n_{\varepsilon }+\rho _{\varepsilon }+\frac{1}{2}$. But $n_{\varepsilon }\le n_{\varepsilon }-\rho _{\varepsilon }+\frac{1}{2}$ and $n_{\varepsilon }+\rho _{\varepsilon }+\frac{1}{2}<n_{\varepsilon }+1$. Therefore $\lfloor x_{\varepsilon }+\frac{1}{2}\rfloor =n_{\varepsilon }$. An analogous argument can be applied to $\text {rni}(-)$. $\square$

Actually, this lemma does not allow us to define a *nearest integer *function  as $\text {ni}{([x_{\varepsilon }])}\,{:=}\,\text {rpi}(x_{\varepsilon })$ because if $[x_{\varepsilon }]=[n_{\varepsilon }]$, the equality $n_{\varepsilon }=\text {rpi}(x_{\varepsilon })$ holds only for $\varepsilon$ small. A simpler approach is to choose a representative $(n_{\varepsilon })\in \mathbb {N}_{\rho }$ for each  and to define $\text {ni}{(x)}\,{:=}\,(n_{\varepsilon })$. Clearly, we must consider the net $\left( \text {ni}{(x)}_{\varepsilon }\right)$ only for $\varepsilon$ small, such as in equalities of the form $x=\left[ \text {ni}{(x)}_{\varepsilon }\right]$. This is what we do in the following

### Definition 17

The *nearest integer function* $\text {ni}(-)$ is defined by:

(i)
(ii) If  and $\text {ni}\left( [x_{\varepsilon }]\right) =(n_{\varepsilon })$ then $\forall ^{0}\varepsilon :\ n_{\varepsilon }=\text {rpi}(x_{\varepsilon })$.

In other words, if , then $x=\left[ \text {ni}(x)_{\varepsilon }\right]$ and $\text {ni}(x)_{\varepsilon }\in \mathbb {N}$ for all $\varepsilon$. Another possibility is to formulate Lemma 16 asTherefore, without loss of generality we may always suppose that $x_{\varepsilon }\in \mathbb {N}$ whenever .

### Remark 18

(i), with the order $\le$ induced by , is a directed set; it is closed with respect to sum and product although recursive definitions using  are not possible.
(ii) In  we can find several chains (totally ordered subsets) such as: $\mathbb {N}$, $\mathbb {N}\cdot [\text {int}(\rho _{\varepsilon }^{-k})]$ for a fixed $k\in \mathbb {N}$, .
(iii) Generally speaking, if *m*, ,  because the net $\left( m_{\varepsilon }^{n_{\varepsilon }}\right)$ can grow faster than any power $(\rho _{\varepsilon }^{-K})$. However, if we take two gauges $\sigma$, $\rho$ satisfying $\sigma \le \rho$, using the net $\left( \sigma _{\varepsilon }^{-1}\right)$ we can measure infinite nets that grow faster than $(\rho _{\varepsilon }^{-K})$ because $\sigma _{\varepsilon }^{-1}\ge \rho _{\varepsilon }^{-1}$ for $\varepsilon$ small. Therefore, we can take *m*,  such that $\left( \text {ni}{(m)}_{\varepsilon }\right)$, $\left( \text {ni}{(n)}_{\varepsilon }\right) \in \mathbb {R}_{\rho }$; we think at *m*, *n* as $\sigma$-hypernatural numbers growing at most polynomially with respect to $\rho$. Then, it is not hard to prove that if $\rho$ is an arbitrary gauge, and we consider the auxiliary gauge $\sigma _{\varepsilon }\,{:=}\,\rho _{\varepsilon }^{e^{1/\rho _{\varepsilon }}}$. then .
(iv) If , then $1^{m}\,{:=}\,\left[ \left( 1+z_{\varepsilon }\right) ^{m_{\varepsilon }}\right]$, where $(z_{\varepsilon })$ is $\rho$-negligible, is well defined and $1^{m}=1$. In fact, $\log (1+z_{\varepsilon })^{m_{\varepsilon }}$ is asymptotically equal to $m_{\varepsilon }z_{\varepsilon }\rightarrow 0$, and this shows that $\left( \left( 1+z_{\varepsilon }\right) ^{m_{\varepsilon }}\right)$ is moderate. Finally, $\left| (1+z_{\varepsilon })^{m_{\varepsilon }}-1\right| \le \left| z_{\varepsilon }\right| m_{\varepsilon }(1+z_{\varepsilon })^{m_{\varepsilon }-1}$ by the mean value theorem.

## Supremum and Infimum in 

To solve the problems we explained in the introduction of this article, it is important to generalize at least two main existence theorems for limits: the Cauchy criterion and the existence of a limit of a bounded monotone sequence. The latter is clearly related to the existence of supremum and infimum, which cannot be always guaranteed in the non-Archimedean ring . As we will see more clearly later (see also [8]), to arrive at these existence theorems, the notion of supremum, i.e. the least upper bound, is not the correct one. More appropriately, we can associate a notion of close supremum (and close infimum) to every topology generated by a set of radii (see Definition 9).

### Definition 19

Let $\mathfrak {R}$ be a set of radii and let $\tau$ be the topology on  generated by $\mathfrak {R}$. Let , then we say that $\tau$ *separates points of* *P* if

$$\begin{aligned} \forall p,q\in P:\ p\mathbb {\ne }q\ \Rightarrow \ \exists A,B\in \tau :\ p\in A,\ q\in B,\ A\cap B=\emptyset , \end{aligned}$$

i.e. if *P* with the topology induced by $\tau$ is Hausdorff.

### Definition 20

Let $\tau$ be a topology on  generated by a set of radii $\mathfrak {R}$ that separates points of  and let . Then, we say that $\sigma$ is $\left( \tau ,P\right)$*-supremum of* *S* if

(i) $$\sigma \in P$$;
(ii) $$\forall s\in S:\ s\le \sigma $$;
(iii) $\sigma$ is a point of closure of *S* in the topology $\tau$, i.e. if $\forall A\in \tau :\ \sigma \in A\ \Rightarrow \ \exists \bar{s}\in S\cap A$.

Similarly, we say that $\iota$ is $(\tau ,P)$-*infimum of S* if

(i) $$\iota \in P$$;
(ii) $$\forall s\in S:\ \iota \le s$$;
(iii) $\iota$ is a point of closure of *S* in the topology $\tau$, i.e. if $\forall A\in \tau :\ \iota \in A\ \Rightarrow \ \exists \bar{s}\in S\cap A$.

In particular, if $\tau$ is the sharp topology and , then following [8], we simply call the $(\tau ,P)$-supremum, the *close supremum* (the adjective close will be omitted if it will be clear from the context) or the *sharp supremum* if we want to underline the dependency on the topology. Analogously, if $\tau$ is the Fermat topology and $P=\mathbb {R}$, then we call the $(\tau ,P)$-supremum the *Fermat supremum*. Note that (iii) implies that if $\sigma$ is $(\tau ,P)$-supremum of *S*, then necessarily $S\ne \emptyset$.

### Remark 21

(i) Let , then from Definition 9 and Theorem 11 we can prove that $\sigma$ is the $(\tau ,P)$-supremum of *S* if and only if
  - $$\forall s\in S:\ s\le \sigma $$;
  - $$\forall r\in \mathfrak {R}\,\exists \bar{s}\in S:\ \sigma -r\le \bar{s}$$.
  In particular, for the sharp supremum, (b) is equivalent to 4.1 In the following of this article, we will also mainly consider the sharp topology and the corresponding notions of sharp supremum and infimum.
(ii) If there exists the sharp supremum $\sigma$ of  and $\sigma \notin S$, then from (4.1) it follows that *S* is necessarily an infinite set. In fact, applying (4.1) with $q_{1}\,{:=}\,1$ we get the existence of $\bar{s}_{1}\in S$ such that . We have $\bar{s}_{1}\ne \sigma$ because $\sigma \notin S$. Hence, Lemma 5.(iii) and Definition 20.(ii) yield that $\bar{s}_{1}<_{\mathrm{s}}\sigma$. Therefore,  for some $q_{2}>q_{1}$. Applying again (4.1) we get  for some $\bar{s}_{2}\in S\setminus \{\bar{s}_{1}\}$. Recursively, this process proves that *S* is infinite. On the other hand, if $S=\{s_{1},\ldots ,s_{n}\}$ and $s_{i}=[s_{i\varepsilon }]$, then $\sup \left( \left[ \{s_{1\varepsilon },\ldots ,s_{n\varepsilon }\}\right] \right) =s_{1}\vee \ldots \vee s_{n}$. In fact, $s_{1}\vee \ldots \vee s_{n}=\left[ \max _{i=1,\ldots ,n}s_{n\varepsilon }\right] \in [\{s_{1\varepsilon },\ldots ,s_{n\varepsilon }\}]$.
(iii) If $\exists \sup (S)=\sigma$, then there also exists the $\sup (\text {interl}(S))=\sigma$, where (see [17]) we recall that  ($1_{S}$ is the characteristic function of $S\subseteq I$). This follows from $S\subseteq \text {interl}(S)$. Vice versa, if $\exists \sup (\text {interl}(S))=\sigma$ and $\text {interl}(S)\subseteq S$ (e.g. if *S* is an internal or strongly internal set), then also $\exists \sup (S)=\sigma$.

### Theorem 22

There is at most one sharp supremum of *S*, which is denoted by $\sup (S)$.

### Proof

Assume that $\sigma _{1}$ and $\sigma _{2}$ are supremum of *S*. That is Definition 20.(ii) and (4.1) hold both for $\sigma _{1}$, $\sigma _{2}$. Then, for all fixed $q\in \mathbb {N}$, there exists $\bar{s}_{2}\in S$ such that . Hence $\bar{s}_{2}\le \sigma _{1}$ because $\bar{s}_{2}\in S$. Analogously, we have that  for some $\bar{s}_{1}\in S$. Therefore, , and this implies $\sigma _{1}=\sigma _{2}$ since $q\in \mathbb {N}$ is arbitrary. $\square$

In [8], the notation $\overline{\sup }(S)$ is used for the close supremum. On the other hand, we will *never* use the notion of supremum as least upper bound. For these reasons, we prefer to use the simpler notation $\sup (S)$. Similarly, we use the notation $\inf (S)$ for the close (or sharp) infimum. From Rem . 21.(a) and (b) it follows that

4.2 $$\begin{aligned} \inf (S)=-\sup (-S) \end{aligned}$$

in the sense that the former exists if and only if the latter exists and in that case they are equal. For this reason, in the following we only study the supremum.

### Example 23

(i) Let  be a functionally compact set (cf. [10]), i.e. $K\subseteq B_{M}(0)$ for some  and $K_{\varepsilon }\Subset \mathbb {R}$ for all $\varepsilon$. We can then define $\sigma _{\varepsilon }\,{:=}\,\sup (K_{\varepsilon })\in K_{\varepsilon }$. From $K\subseteq B_{M}(0)$, we get $\sigma \,{:=}\,[\sigma _{\varepsilon }]\in K$. It is not hard to prove that $\sigma =\sup (K)=\max (K)$. Analogously, we can prove the existence of the sharp minimum of *K*.
(ii) If $S=(a,b)$, where *a*,  and $a\le b$, then $\sup (S)=b$ and $\inf (S)=a$.
(iii) If , then $\inf (S)=0$.
(iv) Like in several other non-Archimedean rings, both sharp supremum and infimum of the set $D_{\infty }$ of all infinitesimals do not exist. In fact, by contradiction, if $\sigma$ were the sharp supremum of $D_{\infty }$, then from (4.1) for $q=1$ we would get the existence of $\bar{h}\in D_{\infty }$ such that . But then $\sigma \in D_{\infty }$, so also $2\sigma \in D_{\infty }$. Therefore, we get $2\sigma \le \sigma$ because $\sigma$ is an upper bound of $D_{\infty }$, and hence , a contradiction. Similarly, one can prove that there does not exist the infimum of this set.
(v) Let $S=\left( 0,1\right) _{\mathbb {R}}=\left\{ x\in \mathbb {R\,}|\,0<x<1\right\}$, then clearly $\sigma =1$ is the Fermat supremum of *S* whereas there does not exist the sharp supremum of *S*. Indeed, if $\sigma =\sup (S)$, then  for all $s\in S$ and for some $\bar{s}\in S$. Taking any $s\in (\bar{s},1)_{\mathbb {R}}\subseteq S$ we get , which, for $\varepsilon \rightarrow 0$, implies $s\le \bar{s}$ because *s*, $\bar{s}\in \mathbb {R}$. This contradicts $s\in (\bar{s},1)$. In particular, 1 is not the sharp supremum. This example shows the importance of Definition 20, i.e. that the best notion of supremum in a non-Archimedean setting depends on a fixed topology.
(vi) Let $S=(0,1)\cup \{\hat{s}\}$ where $\hat{s}|_{L}=2$, $\hat{s}|_{L^{c}}=\frac{1}{2}$, $L\subseteq _{0}I$, $L^{c}\subseteq _{0}I$, then $\not \exists \,\sup (S)$. In fact, if $\exists \sigma \,{:=}\,\sup (S)$, then $\sigma |_{L}\ge \hat{s}|_{L}=2$ and $\sigma |_{L^{c}}=1$. Assume that , then . Thereby, $\bar{s}|_{L}>\frac{3}{2}$ and hence $\bar{s}\not \in (0,1)$ and $\bar{s}=\hat{s}$. We hence get , i.e. , which is impossible. We can intuitively say that the subpoint $\hat{s}|_{L}$ creates a “$\varepsilon$-hole” (i.e. a “hole” only for some $\varepsilon$) on the right of *S* and hence *S* is not “an $\varepsilon$-continuum” on this side. Finally note that the point $u|_{L}\,{:=}\,2$ and $u|_{L^{c}}\,{:=}\,1$ is the least upper bound of *S*.

### Lemma 24

Let *A*, , then

(i), in the sense that one supremum exists if and only if the other one exists, and in that case they coincide;
(ii), in the sense that one supremum/infimum exists if and only if the other one exists, and in that case they coincide;

Moreover, if $\exists \sup (A)$, $\sup (B)$, then:

(iii) If $A\subseteq B$, then $\sup (A)\le \sup (B)$;
(iv) $$\sup (A+B)=\sup (A)+\sup (B)$$;
(v) If *A*, , then $$\sup (A\cdot B)=\sup (A)\cdot \sup (B)$$.

### Proof

(i): If $\exists \sup (\lambda A)$, then we have $a\le \frac{1}{\lambda }\sup (\lambda A)$ for all $a\in A$. For all $q\in \mathbb {N}$, we can find $\bar{a}\in A$ such that . Thereby,  as $q\rightarrow +\infty$ because $\lambda$ is moderate. This proves that $\exists \sup (A)=\frac{1}{\lambda }\sup (\lambda A)$. Similarly, we can prove the opposite implication.

(ii): From (i) and (4.2) we get: $\sup (\lambda A)=\sup (-\lambda (-A))=-\lambda \sup (-A)=\lambda \inf (A)$.

(iii): By contradiction, using Lemma 5.(i), if $\sup (A)>_{L}\sup (B)$ for some $L\subseteq _{0}I$, then  for some $q\in \mathbb {N}$ by Lemma 2 for the ring . Property (4.1) yields  for some $\bar{a}\in A$, and $\bar{a}\le \sup (B)$ because $A\subseteq B$. Thereby, , which implies , a contradiction.

(iv) and (v) follow easily from Definition 20.(ii) and (4.1). $\square$

In the next section, we introduce in the non-Archimedean framework  how to approximate $\sup (S)$ of  using points of *S* and upper bounds, and the non-Archimedean analogous of the notion of upper bound.

## The hyperlimit of a hypersequence

### Definition and examples

#### Definition 25

A map , whose domain is the set of hypernatural numbers  is called a ($\sigma -$) *hypersequence* (of elements of ). The values $x(n)\in$  at  of the function *x* are called *terms* of the hypersequence and, as usual, denoted using an index as argument: $x_{n}=x(n)$. The hypersequence itself is denoted by , or simply $(x_{n})_{n}$ if the gauge on the domain is clear from the context. Let $\sigma$, $\rho$ be two gauges,  be a hypersequence and . We say that *l* is *hyperlimit* of $(x_{n})_{n}$ as $n \rightarrow \infty$ and , ifIn the following, if not differently stated, $\rho$ and $\sigma$ will always denote two gauges and $(x_{n})_{n}$ a $\sigma$-hypersequence of elements of . Finally, if $\sigma _{\varepsilon }\ge \rho _{\varepsilon }$, at least for all $\varepsilon$ small, we simply write $\sigma \ge \rho$.

#### Remark 26

In the assumption of Definition 25, let , $N\in \mathbb {N}$, then the following are equivalent:

(i) is the hyperlimit of $(x_{n})_{n}$ as .
(ii).
(iii) Let  be a sharply open set, if $l\in U$ then .
(iv).
(v).

Directly by the inequality  (or by using that the sharp topology on  is Hausdorff) it follows that there exists at most one hyperlimit, so that we can use the notationAs usual, a hypersequence (not) having a hyperlimit is said to be (non-)convergent. We can also similarly say that  is divergent to $+\infty$ ($-\infty$) if

#### Example 27

(i) If $\sigma \le \rho ^{R}$ for some $R\in \mathbb {R}_{>0}$, we have . In fact,  holds e.g. if  because $\rho _{\varepsilon }^{-q}\le \sigma _{\varepsilon }^{-q/R}$ for $\varepsilon$ small.
(ii) Let $\rho$ be a gauge and set $\sigma _{\varepsilon }\,{:=}\,\exp \bigg (-\rho _{\varepsilon }^{-\frac{1}{\rho _{\varepsilon }}}\bigg )$, so that $\sigma$ is also a gauge. We have  In fact, if $n>1$, we have  if and only if , i.e. (in ). We can thus take  because $e^{\rho _{\varepsilon }^{-q}}<\exp \bigg (\rho _{\varepsilon }^{-\frac{1}{\rho _{\varepsilon }}}\bigg )=\sigma _{\varepsilon }^{-1}$ for $\varepsilon$ small. Vice versa, by contradiction, if , then by the definition of hyperlimit from  to , we would get the existence of  such that 5.1 We have to explore two possibilities: if *l* is not invertible, then $l_{\varepsilon _{k}}=0$ for some sequence $(\varepsilon _{k})\downarrow 0$ and some representative $[l_{\varepsilon }]=l$. Therefore from 25, we get
  $$\begin{aligned} \frac{1}{\log M_{\varepsilon _{k}}}<l_{\varepsilon _{k}}+\rho _{\varepsilon _{k}}=\rho _{\varepsilon _{k}} \end{aligned}$$
  hence $M_{\varepsilon _{k}}>e^{-\frac{1}{\rho _{_{\varepsilon _{k}}}}}$ $\forall k\in \mathbb {N}$, in contradiction with . If *l* is invertible, then  for some $p\in \mathbb {N}$. Setting , we get that $l_{\bar{\varepsilon }_{k}}<\rho _{\bar{\varepsilon }_{k}}^{q}$ for some sequence $(\bar{\varepsilon }_{k})_{k}\downarrow 0$. Therefore
  $$\begin{aligned} \frac{1}{\log M_{\bar{\varepsilon }_{k}}}<l_{\bar{\varepsilon }_{k}}+\rho _{\bar{\varepsilon }_{k}}\le |l_{\bar{\varepsilon }_{k}}|+\rho _{\bar{\varepsilon }_{k}}<\rho _{\bar{\varepsilon }_{k}}^{q}+\rho _{\bar{\varepsilon }_{k}} \end{aligned}$$
  and hence $M_{\bar{\varepsilon }_{k}}>\exp \bigg (\frac{1}{\rho _{\bar{\varepsilon }_{k}}^{q}+\rho _{\varepsilon _{k}}}\bigg )$ for all $k\in \mathbb {N}$, which is in contradiction with  because $q\ge 1$.
  Analogously, we can prove that  if $\sigma =[\sigma _{\epsilon }]=\left[ e^{-e^{\rho _{\epsilon }^{-\frac{1}{\rho _{\epsilon }}}}}\right]$ whereas  (and similarly using $\log (\log (\displaystyle \mathop {\ldots \ldots \,}^{k}(\log n)\ldots )$.
(iii) Set  if $n\in \mathbb {N}$, and $x_{n}\,{:=}\,\frac{1}{n}$ if , then  is unbounded in  even if . Similarly, if  if $n\in \mathbb {N}$ and $x_{n}\,{:=}\,\sin (n)$ otherwise, then $$\lim _{\begin{array}{c} n\rightarrow +\infty \\ n\in \mathbb {N} \end{array} }x_{n}=0$$ whereas . In general, we can hence only state that convergent hypersequence are eventually bounded:
(iv) If $k<_{\mathrm{s}}1$ and $k>_{\mathrm{s}}1$, then  and , hence .
(v) Since for $n\in \mathbb {N}$ we have , it is not hard to prove that  is not a Cauchy sequence. Therefore, , whereas .

A sufficient condition to extend an ordinary sequence  of $\rho$-generalized numbers to the whole  is5.2In fact, in this way  for all , is well-defined because of Lemma 16; on the other hand, we have defined an extension of the old sequence $(a_{n})_{n\in \mathbb {N}}$ because if $n\in \mathbb {N}$, then $\text {ni}(n)_{\varepsilon }=n$ for $\varepsilon$ small and hence $a_{n}=[a_{n}]$. For example, the sequence of infinities  for all $n\in \mathbb {N}$ can be extended to any , whereas  can be extended as  only for some gauges $\rho$, e.g. if the gauges satisfy

5.3 $$\begin{aligned} \exists N\in \mathbb {N}\,\forall n\in \mathbb {N}\,\forall ^{0}\varepsilon :\ \sigma _{\varepsilon }^{n}\ge \rho _{\varepsilon }^{N}, \end{aligned}$$

(e.g. $$\sigma _{\varepsilon }=\varepsilon $$ and $$\rho _{\varepsilon }=\varepsilon ^{1/\varepsilon }$$).

The following result allows us to obtain hyperlimits by proceeding $\varepsilon$-wise

#### Theorem 28

Let $(a_{n,\varepsilon })_{n,\varepsilon }:\mathbb {N}\times I\longrightarrow \mathbb {R}$. Assume that for all $\varepsilon$

5.4 $$\begin{aligned} \exists \lim _{n\rightarrow +\infty }a_{n,\varepsilon }{=}{:}l_{\varepsilon }, \end{aligned}$$

and that . Then there exists a gauge $\sigma$ (not necessarily a monotonic one) such that

(i) There exists  and a hypersequence  such that  for all ;
(ii).

#### Proof

From (5.4), we have

5.5 $$\begin{aligned} \forall \varepsilon \,\forall q\,\exists M_{\varepsilon q}\in \mathbb {N}_{>0}\,\forall n\ge M_{\varepsilon q}:\ \rho _{\varepsilon }^{q}-l_{\varepsilon }<a_{n,\varepsilon }<\rho _{\varepsilon }^{q}+l_{\varepsilon }. \end{aligned}$$

Without loss of generality, we can assume to have recursively chosen $M_{\varepsilon q}$ so that

5.6 $$\begin{aligned} M_{\varepsilon q}\le M_{\varepsilon ,q+1}\quad \forall \varepsilon \,\forall q. \end{aligned}$$

Set $\bar{M}_{\varepsilon }\,{:=}\,M_{\varepsilon ,\lceil \frac{1}{\varepsilon }\rceil }>0$; since $\forall q\in \mathbb {N}\,\forall ^{0}\varepsilon :\ q\le \lceil \frac{1}{\varepsilon }\rceil$, (5.6) implies

5.7 $$\begin{aligned} \forall q\in \mathbb {N}\,\forall ^{0}\varepsilon :\ \bar{M}_{\varepsilon }\ge M_{\varepsilon q}. \end{aligned}$$

If the net $(\bar{M}_{\varepsilon })$ is $\rho$-moderate, set $\sigma \,{:=}\,\rho$, otherwise set $\sigma _{\varepsilon }\,{:=}\,\min \left( \rho _{\varepsilon },\bar{M}_{\varepsilon }^{-1}\right) \in (0,1]$. Thereby, the net $\sigma _{\varepsilon }\rightarrow 0$ as $\varepsilon \rightarrow 0^{+}$ (note that not necessarily $\sigma$ is non-decreasing, e.g. if $\lim _{\varepsilon \rightarrow \frac{1}{k}}\bar{M}_{\varepsilon }=+\infty$ for all $k\in \mathbb {N}_{>0}$ and $\bar{M}_{\varepsilon }\ge \rho _{\varepsilon }^{-1}$), i.e. it is a gauge. Now set  because our definition of $\sigma$ yields $\bar{M}_{\varepsilon }\le \sigma _{\varepsilon }^{-1}$,  because of (5.7), and5.8We have to prove that this well-defines a hypersequence . First of all, the sequence is well-defined with respect to the equality in  because of Lemma 16. Moreover, setting $q=1$ in (5.5), we get $\rho _{\varepsilon }-l_{\varepsilon }<a_{n,\varepsilon }<\rho _{\varepsilon }+l_{\varepsilon }$ for all $\varepsilon$ and for all $n\ge M_{\varepsilon 1}$. If $n\ge M_{1}$ in , then $\text {ni}{(n)}_{\varepsilon }\ge M_{\varepsilon 1}$ for $\varepsilon$ small, and hence $\rho _{\varepsilon }-l_{\varepsilon }<a_{\text {ni}{(n)}_{\varepsilon },\varepsilon }<\rho _{\varepsilon }+l_{\varepsilon }$. This shows that  because we assumed that . Finally, (5.5) and (5.6) yield that if $n\ge M_{q}$ then $n\ge M_{1}$ and hence . $\square$

From the proof it also follows, more generally, that if $(M_{\varepsilon q})_{\varepsilon ,q}$ satisfies (5.5) and if

$$\begin{aligned} \exists (q_{\varepsilon })\rightarrow +\infty :\ \left( M_{\varepsilon ,q_{\varepsilon }}\right) \in \mathbb {R}_{\rho }, \end{aligned}$$

then we can repeat the proof with $q_{\varepsilon }$ instead of $\lceil \frac{1}{\varepsilon }\rceil$ and setting $\sigma \,{:=}\,\rho$.

### Operations with hyperlimits and inequalities

Thanks to Definition 9 of sharp topology and our notation for $x<y$ (and of the consequent Lemma 2), some results about hyperlimits can be proved by trivially generalizing classical proofs. For example, if  and  are two convergent hypersequences then their sum , product  and quotient  (the last one being defined only when $y_{n}$ is invertible for all ) are convergent hypersequences and the corresponding hyperlimits are sum, product and quotient of the corresponding hyperlimits.

The following results generalize the classical relations between limits and inequalities.

#### Theorem 29

Let *x*, *y*,  be hypersequences, then we have:

(i) If , then  such that $x_{n}<y_{n}$ for all $n\ge M$, .
(ii) If $x_{n}\le y_{n}\le z_{n}$ for all  and , then

#### Proof

(i) follows from Lemma 2 and the Definition 25 of hyperlimit. For (ii), the proof is analogous to the classical one. In fact, since  given $q\in \mathbb {N}$, there exist $M'$,  such that  and  for all $n>M'$, , then for $n>M\,{:=}\,M'\vee M''$, we have . $\square$

#### Theorem 30

Assume that *C* is a sharply closed subset of , that  and that $x_{n}$ eventually lies in *C*, i.e. . Then also $l\in C$. In particular, if $(y_{n})_{n}$ is another hypersequence such that , then  implies $l\ge k$.

#### Proof

A reformulation of the usual proof applies. In fact, let us suppose that . Since  is sharply open, there is an $\eta >0,$ for which . Let  be such that $|x_{n}-l|<\eta$ when $n>\bar{n}$. Then we have $x_{n}\in C$ and , a contradiction. $\square$

The following result applies to all generalized smooth functions (and hence to all Colombeau generalized functions, see e.g. [11, 12]; see also [1] for a more general class of functions) because of their continuity in the sharp topology.

#### Theorem 31

Suppose that . Then *f* is sharply continuous function at $x=c$ if and only if it is hyper-sequentially continuous, i.e. for any hypersequence $\left( x_{n}\right) _{n}$ in *U* converging to *c*, the hypersequence $\left( f\left( x_{n}\right) \right) _{n}$ converges to $f\left( c\right)$, i.e. .

#### Proof

We only prove that the hyper-sequential continuity is a sufficient condition, because the other implication is a trivial generalization of the classical one. By contradiction, assume that for some $Q\in \mathbb {N}$5.9For $n\in \mathbb {N}$ set $\omega _{n}\,{:=}\,n$ and for  set  and $x_{n}\,{:=}\,x_{\omega _{n}}$. Then for all , from (5.9) we get  because $\omega _{n}\rightarrow +\infty$ as $n\rightarrow +\infty$ in . Therefore, $(x_{n})_{n}$ is an hypersequence of *U* that converges to *c*, which yields $f(x_{n})\rightarrow f(c)$, in contradiction with (5.9). $\square$

#### Example 32

Let $\sigma \le \rho ^{R}$ for some $R\in \mathbb {R}_{>0}$. The following inequalities hold for all generalized numbers because they also hold for all real numbers:

5.10 $$\begin{aligned} \ln (x)&\le x\nonumber \\ e\left( \frac{n}{e}\right) ^{n}&\le n!\le en\left( \frac{n}{e}\right) ^{n}. \end{aligned}$$

From the first one it follows $0\le \frac{\ln (n)}{n}=\frac{2\ln \sqrt{n}}{n}\le \frac{2\sqrt{n}}{n}$, so that  from Theorem 29 and  from Theorem 31 and hence  by (5.10). Similarly, we have  because $n\log \left( 1+\frac{1}{n}\right) =1-\frac{1}{2n}+O\left( \frac{1}{n^{2}}\right) \rightarrow 1$ and because of Theorem 31.

A little more involved proof concerns L’Hôpital rule for generalized smooth functions. For the sake of completeness, here we only recall the equivalent definition:

#### Definition 33

Let  and . We say that $f:X\longrightarrow Y$ is a *generalized smooth function* (GSF) if

(i) $f:X\longrightarrow Y$ is a set-theoretical function.
(ii) There exists a net $(f_{\varepsilon })\in {{\mathcal {C}}}^{\infty }(\mathbb {R}^{n},\mathbb {R}^{d})^{(0,1]}$ such that for all $[x_{\varepsilon }]\in X$:
  - $$f(x)=[f_{\varepsilon }(x_{\varepsilon })]$$
  - $$\forall \alpha \in \mathbb {N}^{n}:\ (\partial ^{\alpha }f_{\varepsilon }(x_{\varepsilon }))\text { is }\rho -\text {moderate}$$.

For generalized smooth functions lots of results hold: closure with respect to composition, embedding of Schwartz’s distributions, differential calculus, one-dimensional integral calculus using primitives, classical theorems (intermediate value, mean value, Taylor, extreme value, inverse and implicit function), multidimensional integration, Banach fixed point theorem, a Picard-Lindelöf theorem for both ODE and PDE, several results of calculus of variations, etc.

In particular, we have the following (see also [9] for the particular case of Colombeau generalized functions):

#### Theorem 34

Let  be a sharply open set and let  be a GSF defined by the net of smooth functions $f_{\varepsilon }\in {{\mathcal {C}}}^\infty (\mathbb {R},\mathbb {R})$. Then

(i) There exists an open neighbourhood *T* of $U\times \{0\}$ and a GSF , called the *generalized incremental ratio* of *f*, such that
  5.11 $$\begin{aligned} f(x+h)=f(x)+h\cdot R_{f}(x,h)\qquad \forall (x,h)\in T. \end{aligned}$$
  Moreover $R_{f}(x,0)=\left[ f'_{\varepsilon }(x_{\varepsilon })\right] =f'(x)$ is another GSF and we can hence recursively define $f^{(k)}(x)$.
(ii) Any two generalized incremental ratios of *f* coincide on the intersection of their domains.
(iii) More generally, for all $k\in \mathbb {N}_{>0}$ there exists an open neighbourhood *T* of $U\times \{0\}$ and a GSF , called *k*-*th order Taylor ratio*of *f*, such that
  5.12 $$\begin{aligned} f(x+h)=\sum _{j=0}^{k-1}\frac{f^{(j)}(x)}{j!}h^{j}+R_{f}^{k}(x,h)\cdot h^{k}\qquad \forall (x,h)\in T. \end{aligned}$$
  Any two ratios of *f* of the same order coincide on the intersection of their domains.

We can now prove the following generalization of one of L’Hôpital rule:

#### Theorem 35

Let  be a sharply open set $(x_{n})_{n}$,  be hypersequences converging to $l\in U$ and $m\in U$ respectively and such thatLet $k\in \mathbb {N}_{>0}$ and *f*,  be GSF such that for all  and all $j=0,\ldots ,k-1$5.13Then for all $j=0,\ldots ,k-1$

#### Proof

Using (5.12) and (5.13), we can write

$$\begin{aligned} \frac{f(x_{n})}{g(y_{n})}= & {} \frac{\sum _{j=0}^{k-1}\frac{f^{(j)}(l)}{j!}(x_{n}-l)^{j}+(x_{n}-l)^{k}R_{f}^{k}(l,x_{n}-l)}{\sum _{j=0}^{k-1}\frac{g^{(j)}(m)}{j!}(y_{n}-m)^{j}+(y_{n}-m)^{k}R_{g}^{k}(m,y_{n}-m)}\\= & {} \left( \frac{x_{n}-l}{y_{n}-m}\right) ^{k}\cdot \frac{R_{f}^{k}(l,x_{n}-l)}{R_{g}^{k}(m,y_{n}-m)}. \end{aligned}$$

Since $R_{f}^{k}$ and $R_{g}^{k}$ are GSF, they are sharply continuous. Therefore, the right hand side of the previous equality tends to $C^{k}\cdot \frac{R_{f}^{k}(l,0)}{R_{g}^{k}(m,0)}=C^{k}\cdot \frac{f^{(k)}(l)}{g^{(k)}(m)}$. At the same limit converges the quotient $C^{k}\frac{f^{(k)}(x_{n})}{g^{(k)}(y_{n})}$ because $f^{(k)}$ and $g^{(k)}$ are also GSF and hence they are sharply continuous. The claim for $j=1,\ldots ,k-1$ follows by applying the conclusion for $j=0$ with $f^{(j)}$ and $g^{(j)}$ instead of *f* and *g*. $\square$

Note that for $x_{n}=y_{n}$, $l=m$, we have $C=1$ and we get the usual L’Hôpital rule (formulated using hypersequences). Note that a similar theorem can also be proved without hypersequences and using the same Taylor expansion argument as in the previous proof.

### Cauchy criterion and monotonic hypersequences.

In this section, we deal with classical criteria implying the existence of a hyperlimit.

#### Definition 36

We say that  is a *Cauchy hypersequence* if

#### Theorem 37

A hypersequence converges if and only if it is a Cauchy hypersequence

#### Proof

To prove that the Cauchy criterion is a necessary condition it suffices to consider the inequalities:Vice versa, assume that5.14The idea is to use Cauchy completeness of . In fact, set $h_{1}\,{:=}\,M_{1}$ and $h_{q+1}\,{:=}\,M_{q+1}\vee h_{q}$. We claim that $(x_{h_{q}})_{q\in \mathbb {N}}$ is a standard Cauchy sequence converging to the same limit of  . From (5.14) it follows that $(x_{h_{q}})_{q\in \mathbb {N}}$ is a standard Cauchy sequence (in the sharp topology). Therefore, there exists  such that $\lim _{q\rightarrow +\infty }x_{h_{q}}=\bar{x}$. Now, fix $q\in \mathbb {N}$ and pick any $m\ge q+1$ such that5.15Then for all $N\ge M_{q+1}$ we have:because $h_{m}\ge h_{q+1}\ge M_{q+1}$ so that we can apply (5.14) and (5.15). $\square$

#### Theorem 38

A hypersequence converges if and only if

#### Proof

It suffices to apply the inequality $\left| x_{n}-x_{m}\right| \le \left| x_{n}-x_{n\vee m}\right| +\left| x_{n\vee m}-x_{m}\right|$. $\square$

The second classical criterion for the existence of a hyperlimit is related to the notion of monotonic hypersequence. The existence of several chains in  does not allow to arrive at any  starting from any other lower  and using the successor operation only a finite number of times. For this reason, the following is the most natural notion of monotonic hypersequence:

#### Definition 39

We say that  is a *non-decreasing* (or *increasing*) hypersequence ifSimilarly, we can define the notion of non-increasing (decreasing) hypersequence.

#### Theorem 40

Let  be a non-decreasing hypersequence. Thenand in that case they are equal.

#### Proof

Assume that  converges to *l* and set , we will show that $l=\sup (S)$. Now, using Definition 25, we have that  for some . But from Definition 39. Therefore  for all , and the conclusion $x_{n}\le l$ follows since $q\in \mathbb {N}$ is arbitrary. Finally, from Definition 25 of hyperlimit, for all $q\in \mathbb {N}$ we have the existence of  such that  which completes the necessity part of the proof. Now, assume that $\exists \,\sup (S){=}{:}l$. We have to prove that . In fact, using Rem. (i), we getand  for all  by Definition 39 of monotonicity. That is, . $\square$

#### Example 41

The hypersequence  is non-decreasing. Assume that $(x_{n})_{n}$ converges to *l* and that $\sigma \le \rho ^{R}$ for some $R\in \mathbb {R}_{>0}$. Since , by Theorem 30, we get . Therefore, applying the logarithm and the exponential functions, from Theorem 31 we obtain that $l=1$ because from $\sigma \le \rho ^{R}$ it follows that . But this is impossible since . Thereby, .

## Limit superior and inferior

We have two possibilities to define the notions of limit superior and inferior in a non-Archimedean setting such as : the first one is to assume that both  and  exist (the former for all ); the second possibility is to use inequalities to avoid the use of supremum and infimum. In fact, in the real case we have $\iota \le \sup _{n\ge m}x_{n}\le \iota +\varepsilon$ if and only if

$$\begin{aligned}&\forall n\ge m:\ x_{n}\le \iota +\varepsilon \\&\forall \varepsilon \,\exists \bar{n}\ge m:\ \iota -\varepsilon \le x_{\bar{n}}. \end{aligned}$$

### Definition 42

Let  be an hypersequence, then we say that  is the *limit superior* of $(x_{n})_{n}$ if

(i);
(ii).

Similarly, we say that  is the *limit inferior* of $(x_{n})_{n}$ if

(iii);
(iv).

We have the following results (clearly, dual results hold for the limit inferior):

### Theorem 43

Let $(x_{n})_{n}$,  be hypersequences, then

(i) There exists at most one limit superior and at most one limit inferior. They are denoted with  and .
(ii) If  for all , then  if and only if , and in that case
(iii) in the sense that if one of them exists, then also the other one exists and in that case they are equal.
(iv) if and only if .
(v) If , , , then  In particular, if , then the existence of the single limit superiors implies the existence of the limit superior of the sum.
(vi) If $x_{n}$, $y_{n}\ge 0$ for all  and if , , , then  In particular, if , then the existence of the single limit superiors implies the existence of the limit superior of the product.
(vii) If , then there exists a sequence $(\bar{n}_{q})_{q\in \mathbb {N}}$ of  such that
  - $$\bar{n}_{q+1}>\bar{n}_{q}$$ for all $$q\in \mathbb {N}$$;
  - $$\lim _{q\rightarrow +\infty }\bar{n}_{q}=+\infty $$ in ;
  - $$\exists \lim _{q\rightarrow +\infty }x_{\bar{n}_{q}}=\iota $$.
(viii) Assume to have a sequence $(\bar{n}_{q})_{q\in \mathbb {N}}$ satisfying the previous conditions (a), (b), (c) and 6.1 Then .

### Proof

(i): Let $\iota _{1}$, $\iota _{2}$ be both limit superior of $(x_{n})_{n}$. Based on Lemma 6.(iii), without loss of generality we can assume that $\iota _{1}<_{\mathrm{s}}\iota _{2}$. According to Lemma 2, there exists $m\in \mathbb {N}$ such that . Take $q_{1}$, $q_{2}$ large enough so that . Using the last two inequalities, we obtain6.2Using Definition 42.(i), we can find  such that6.3Using Definition 42.(ii) with $q=q_{1}$ and $N=N_{1}$, we get6.4We now use (6.2), (6.4) and (6.3) for $n=\bar{n}$ and we obtain , which is a contradiction.

(ii): Lemma 24. (iii) implies that $(\alpha _{m})_{m}$ is non-increasing. Therefore, we have  if these terms exist from Theorem 40. But Cor. ?? and Definition 42.(i) imply . Finally, Definition 42.(ii) yields , which proves that .

(iii): Directly from Definition 42.

(iv): Assume that hyperlimit superior and inferior exist and are equal to *l*. From Definition 42.(i) and Definition 42.(iii) we get  for all $n\ge N$. Vice versa, assume that the hyperlimit exists and equals *l*, so that  for all $n\ge N$. Then both Definition 42.(i) and Definition 42.(iii) trivially hold. Finally, Definition 42.(ii) and Definition 42.(iv) hold taking e.g. $\bar{n}=N$.

(v): Settingfrom Definition 42 we get , which implies $l\le \iota +j$ for $q\rightarrow +\infty$. Adding Definition 42.(ii) we obtain  for some $\bar{n}$, . Therefore, if $x_{\bar{n}}+y_{\hat{n}}\le x_{n}+y_{n}$ for some $n\ge N$, this yields the second claim. Similarly, one can prove (vi).

(vii): From Definition 42.(i), choose an $N_{q}=N$ for each $q\in \mathbb {N}$, i.e.6.5Applying Definition 42.(ii) with $q>0$ and , we get the existence of $\bar{n}_{q}\ge N_{q}$ such that both (a) and (b) hold and . Thereby, from (6.5) we also get (c).

(viii): Write (c) as6.6Set . For $n\ge N$, from (6.1) we get the existence of $p\in \mathbb {N}$ such that $\bar{n}_{p}\ge n$ and $x_{n}\le x_{\bar{n}_{p}}$. Thereby, $\bar{n}_{p}\ge \bar{n}_{Q_{q}}$ and hence $p\ge Q_{q}$ because of (a) and thus . Finally, condition (ii) of Definition 42 follows from (6.6) and (b). $\square$

It remains an open problem to show an example that proves as necessary the assumption of Theorem 43.(ii), i.e. that that the previous definition of limit superior and inferior is strictly more general than the simple transposition of the classical one.

### Example 44

(i) Directly from Definition 42, we have that
(ii) Let  be such that $\mu |_{L}=1$ and $\mu |_{L^{c}}=-1$, where *L*, $L^{c}\subseteq _{0}I$. Then $\mu ^{n}\le 1$ and  if $\text {ni}{(\bar{n})}_{\varepsilon }$ is even for all $\varepsilon$ small. Therefore , whereas .
(iii) From (vii) and (viii) of Theorem43 it follows that for an increasing hypersequence $(x_{n})_{n}$,  if and only if . Therefore, example 41 implies that .

## Conclusions

In this work we showed how to deal with several deficiencies of the ring of Robinson-Colombeau generalized numbers : trichotomy law for the order relations $\le$ and <, existence of supremum and infimum and limits of sequences with a topology generated by infinitesimal radii. In each case, we obtain a faithful generalization of the classical case of real numbers. We think that some of the ideas we presented in this article can inspire similar works in other non-Archimedean settings such as (constructive) nonstandard analysis, p-adic analysis, the Levi-Civita field, surreal numbers, etc. Clearly, the notions introduced here open the possibility to extend classical proofs in dealing with series, analytic generalized functions, sigma-additivity in integration of generalized functions, non-Archimedean functional analysis, just to mention a few.
